# First Steps into the Wild – Exploration Behavior of European Bison after the First Reintroduction in Western Europe

**DOI:** 10.1371/journal.pone.0143046

**Published:** 2015-11-25

**Authors:** Philip Schmitz, Stephanie Caspers, Paige Warren, Klaudia Witte

**Affiliations:** 1 Institute of Biology, Research Group of Ecology and Behavioral Biology, University of Siegen, Siegen, Germany; 2 Department of Environmental Conservation, University of Massachusetts, Amherst, Massachusetts, United States of America; Auburn University, UNITED STATES

## Abstract

Biodiversity is rapidly declining globally. One strategy to help to conserve species is to breed species in captivity and release them into suitable habitats. The way that reintroduced animals explore new habitats and/or disperse from the release site is rarely studied in detail and represents key information for the success of reintroduction projects. The European bison (*Bison bonasus* L. 1758) was the largest surviving herbivore of the post-glacial megafauna in Europe before it became extinct in the wild, surviving only in captivity since 1919. We investigated the exploration behavior of a herd of European bison reintroduced into the Rothaargebirge, a commercial forest in low range mountain intensively used and densely populated by humans, in the first six months after release. We focused on three questions: (1) how did the European bison move and utilize the habitat on a daily basis, (2) how did the animals explore the new environment, and (3) did their habitat preferences change over time. The European bison dispersed away from their previous enclosure at an average rate of 539 m/month, with their areas of daily use ranging from 70 to 173 ha, their movement ranging from 3.6 km to 5.2 km per day, and their day-to-day use of areas ranged between 389 and 900 m. We could identify three major exploration bouts, when the animals entered and explored areas previously unknown to them. During the birthing phase, the European bison reduced daily walking distances, and the adult bull segregated from the herd for 58 days. Around rut, roaming behavior of the herd increased slightly. The animals preferred spruce forest, wind thrown areas and grassland, all of which are food abundant habitat types, and they avoided beech forest. Habitat preference differed slightly between phases of the study period, probably due to phenological cycles. After six months, the complete summer home range was 42.5 km^2^. Our study shows that a small free-ranging herd of European bison can live in an area intensively used by humans and describes in detail the initial roaming behavior and habitat utilization of the animals.

## Introduction

Biodiversity is rapidly declining globally. Out of the 61,898 taxa listed by the International Union for Conservation of Nature and Natural Resources (IUCN), 35.9% are either extinct in the wild (0.1%), critically endangered (7.4%), endangered (11.0%), or vulnerable (17.4%), and thus referred to as threatened species [1]. Of these threatened animals, 22.1% are mammals, including such large herbivorous species as the European bison (*Bison bonasus*, L. 1758). Most species became endangered due to overexploitation, habitat loss, invasive species and the synergistic effects of these factors [2], [3], [4], [5], [6], [7].

Commonly recommended strategies for preventing the loss of critically endangered species include *ex situ* approaches (protecting endangered species outside their natural habitats) and *in situ* approaches (protecting a species within its natural habitat), or a combination of both strategies by reintroducing captive bred animals to suitable habitats and to restore and connect populations [8], [9], [10]. Fragmented habitats can be connected by establishing (sub)-populations that work as corridors, thus increasing population size and gene flow, minimizing the risk of local extinction and promoting the species' survival [11], [12], [13]. Although reintroductions involve many logistical and financial challenges [14] they have been successfully carried out numerous times [6]. Examples include the Przewalski's horses (*Equus ferus przewalskii*) in Mongolia [15], the Arabian Oryx (*Oryx leucoryx*) in Saudi Arabia and Israel [16], [17], the European bison (*Bison bonasus*) in several eastern countries [11], [18], [19], the Alpine ibex (*Capra ibex ibex*) in the European Alps [20], the Black bear (*Ursus americanus*) in Arkansas [21], the River otter (*Lontra canadensis*) in Pennsylvania [22], the Bearded vulture (*Gypaetus barbatus*) in the European alps [23], the Yellow-Shouldered Amazon Parrots (*Amazona barbadensis*) in Venezuela [24], and the Baton Blue butterfly (*Pseudophilotes baton schiffermuelleri*) in Finland [25].

Because species considered for reintroduction may lack their original habitat types or lack unaltered habitat (e.g., due to human land use), it is essential for successful reintroductions to find suitable environments that can meet a species’ habitat requirements [10]. Additionally, captive-bred animals must be able to develop natural behavior and acclimate to the new habitat. Therefore, reintroduction processes require a detailed knowledge of the animal's biology and behavior prior to and detailed monitoring after reintroduction [26]. In particular, knowledge of how animals explore the new habitat and whether they disperse from the release site are key pieces of information for evaluating the success of a reintroduction and for conflict management in human dimensions, especially in large animals [27], [28], [29]. Despite a rich publication record dealing with animal reintroductions, only a few detailed descriptions of newly reintroduced animals documenting the first dispersal from their release site are available [23], [24], [30]. Information about such processes and especially the exploration behavior of a reintroduced species are very important for further reintroduction programs and should carefully be investigated and documented.

The European bison is, aside from the now extinct Aurochs (*Bos primigenius* Bojanus, 1827), the largest surviving post-glacial megaherbivore in Western Europe. The species was extirpated in the wild in 1927 [11], but persisted due to a small captive breeding population [18]. Today, free roaming European bison live in 35 populations across seven countries. The world's population consists of 4,987 animals; 3,102 (62%) of these as free-ranging in eastern European countries [19], [31].

European bison were mainly reintroduced to forest ecosystems since their last appearance was in such areas (Białowieża primeval forest and Caucasus) and were commonly classified as forest specialists [11], [32]. In recent years, this classification has been revised according to the refugee species concept [33], [34], [35], [36]. Considerable evidence has emerged that European bison are adapted to live in semi-open and mosaic-like habitats and were pushed into forest ecosystems by anthropogenic influences such as habitat loss and fragmentation, competition with livestock, diseases, hunting or poaching [11], [33], [34], [35], [36]. Today, there is almost no landscape without any anthropogenic influence left in Western Europe. Thus, the reintroduction of species faces the dual challenge of human imposed constraints and finding suitable areas for species whose original habitats are gone. As a result it is important to understand the animals' exploration behavior and ability to recolonize modified habitats as part of the reintroduction process [11], [29]. The first reintroduction of European bison into Western Europe has been carried out in a commercial forest in the Rothaargebirge in Germany.

We investigated the exploration behavior of the released herd for the first six months following reintroducing. We focused on three main questions to better understand the exploration behavior of a newly reintroduced large herbivore: (1) how did the European bison move and use habitat on a daily basis, (2) how did the animals explore the new environment, and (3) did their habitat preferences change over time.

## Material and Methods

### Study species

**Fig 1:**
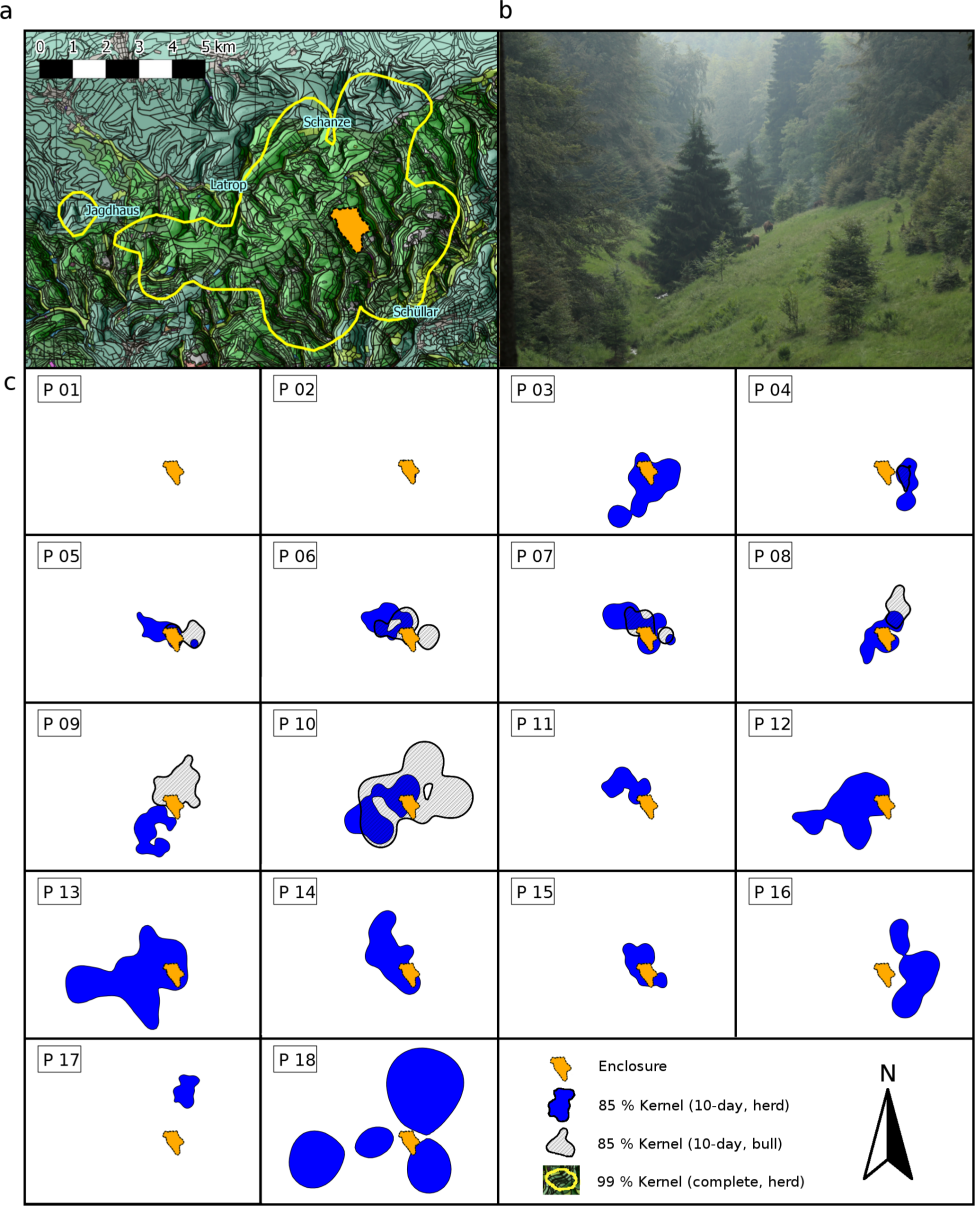
Exploration of the new habitat in the European bison during the first six month after release into the wild. (a) The overall used area (99% kernel h_ref_ of the herd after six months). (b) Typical habitat frequently used by the European bison in the Rothaargebirge. (c) Used areas (85% kernel h_ref_) of the herd and the solitary roaming bull of each 10-days period (P1-P18). The former enclosure is indicated by the orange area. Three major exploration bouts were observed in P3, P13 and P18, when the animals entered new areas.

Since April 2010, a herd of European bison, obtained from different zoos in Germany and Belgium, were kept in an 89 ha enclosure within the Rothaargebirge (Gauss-Krueger coordinates: 3456254, 5663510) centrally located in the study area as described above. During a three-year period between 2010 and 2013 we intensively studied ethology and the habituation process of the animals and their impact on the area [37], [38], [39], [40], [41], [42], [43], [44]. A single herd of eight animals (1 bull, 6 cows, 1 calf, Table 1) was released into the commercial forest using a soft-release method by removing the fence on 11th April 2013. This was the first reintroduction of this species in Western Europe (Fig 1A and 1B) [45].

**Table 1:** European bison released into the wild on 11^th^ April 2013. Pedigree numbers are listed in front of names. (*) Pedigree number not yet assigned.

Pedigree No, Name	Sex	Date of birth	Heritage	Father	Mother
11337 Abdia	f	17.09.2008	Bayerischer Wald	9737 Abkes	9701 Abdil
11336 Abtisa	f	09.10.2008	Bayerischer Wald	9737 Abkes	9186 Abtei
10661 Araneta	f	28.09.2006	Amsterdam	7642 Dudo	7636 Kreole
11303 Dareli	f	28.09.2008	Damerower Werder	8741 Eggedämon	10203 Dara
11347 Daviedi	f	08.12.2008	Damerower Werder	9570 Daaks	7064 Danica
10754 Egnar	m	22.09.2006	Hardehausen I	9583 Kuabo	9166 Eglaja
12044 Queen	f	17.08.2011	Bad Berleburg	11338 Horno	10661 Araneta
12272 Quandor	m	24.06.2012	Bad Berleburg	10754 Egnar	11303 Dareli
QU_05 *	m	05.05.2013	Bad Berleburg	10754 Egnar	10661 Araneta
QU_06 *	m	23.05.2013	Bad Berleburg	10754 Egnar	11337 Abdia

### Study area

The area for the reintroduction was in the Rothaargebirge, Germany, in a low mountain range area located between 450 m and 750 m above sea level [45]. It was an intensively used commercial forest with a mosaic of open areas, meadows and mostly monoculture forest plantations, in the most densely populated federal state of Germany (North-Rhine Westphalia) with 523.4 inhabitants / km^2^ and additional 1.5 million tourists visiting the area annually [29], [46], [47]. The area covered over 4,500 ha of private commercial forest, consisting of spruce forest (*Picea abies*, 44.4%), beech forest (*Fagus sylvatica*, 33.7%, e.g. *Luzulo-Fagetum*), oak forest (*Quercus robur*, 0.4%), douglas fir forest (*Pseudotsuga menziesii*, 0.4%), larch forest (*Larix sp*., 0.2%), other deciduous forest (0.6%), storm damaged areas (succession zones, e.g. *Digitalis purpurea-Epilobium angustifolium-*association, 4.4%), grasslands (8.7%), roads (6.9%), many creeks, and other land use (settlements and pine forests, 0.3%, Fig 1B). The herbaceous biomass and available nutritional energy per hectare in the study area was unevenly distributed among the forest types [48]. Ungulate game species occurring in this area included wild boar (*Sus scrofa*), mouflon sheep (*Ovis orientalis musimon*), roe deer (*Capreolus capreolus*) and red deer (*Cervus elaphus*). Sightings of single European lynx (*Lynx lynx*) and wolves (*Canis lupus*) were reported, but these animals did not regularly occur in the Rothaargebirge [49]. The study area was demarcated in the south by a game fence, built some 25 years ago (Gauss-Krueger coordinates: southernmost point: 3452862, 5657882). The western border was the street K42 (westernmost point: 3448397, 5662446) and the eastern border the street B480 (easternmost point: 3460432, 5662412). To the north it was demarcated by the ridge of the Rothaargebirge and the hiking trail “Rothaarsteig” (northermost point: 3459880, 5667353). There was no physical border aside from the game fence in the south part of the reintroduction area. Centrally located in this study area was an enclosure (now removed) of 89 ha in which the herd lived for three years (2010–2013) before release with a feeding site. Concentrated feed (corn, wheat, dried beet slices) was given in small amounts (ca. 2.5 to 5.0 kg) several times per week at irregular intervals.

### Data analyses

#### General procedure

**Fig 2:**
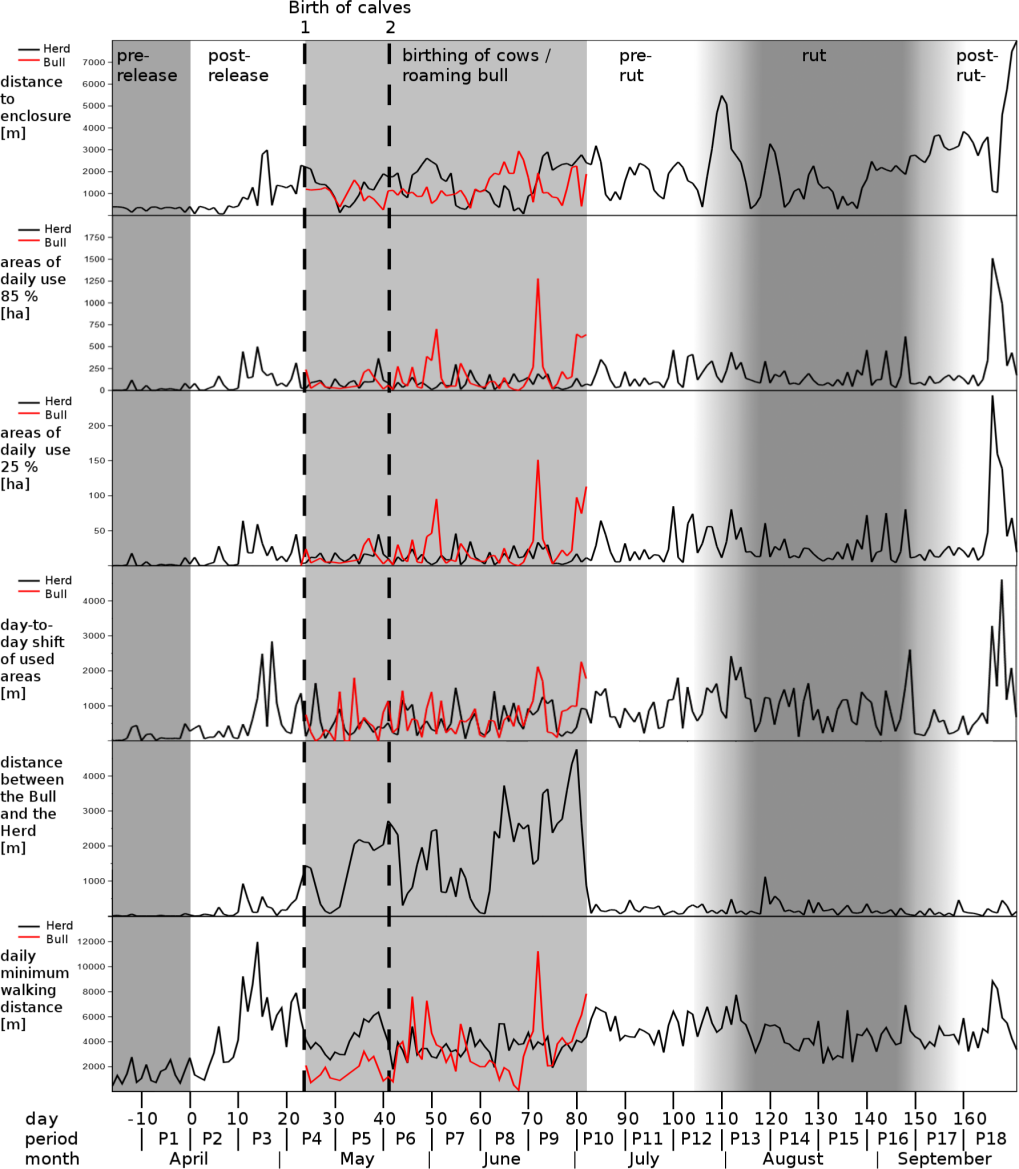
Overview of our measurements during the eighteen 10-days-periods assigned to six different phases (pre-/ post-release, birthing / roaming bull, pre-rut, rut, post-rut). Distinct phases are indicated by underlying grey bars. The births of the calves are indicated by dotted lines. We distinguished between the bull (red line) and herd (black line) for most measurements between day 24 and 82 (P4-P10).

We divided the six month observation period (2nd April–30th September) into 18 periods of 10 days each (P1—P18, Fig 1C, Fig 2) starting with 10 days prior to release (P1).

Based on the release date and the behavior of European bison we assigned the 10-day-periods to six different phases: pre-release, post release, birthing, pre-rut, rut, and post-rut and the separation and reunion of the adult bull to the herd (Table 2, Fig 2, S1A–S1E Table) and calculated medians, and the first and third quartiles, for each phase to give a general overview of the changes in behavior. All geographical analyses were carried out using Quantum GIS 1.8 [50]. Statistical analyses were calculated using PAST 3.0 [51]. We used a significance level of P < 0.05 for each test.

**Table 2:** Description of phases.

Phase	Days	Date	Description
Pre-release	-10 to 0	02.04.2013 to 11.04.2013	Animals were kept inside the enclosure (management).
Post-release	1 to 23	12.04.2013 to 05.05.2013	Animals were released into the Rothaargebirge (management).
Birthing of cows / solitary roaming bull	24 to 82	06.05.2013 to 03.07.2013	The bull was mainly separated from the herd. Two calves were delivered.
Pre-rut	83 to 108	04.07.2013 to 29.07.2013	Period between the adult bull's return to the herd and the rutting season.
Rut	109 to 155	30.7.2013 to 14.09.2013	The rutting season was determined following [18], [54], [71], [73] [77], [78]. Additional direct observation revealed the occurrence of rutting behavior in the herd.
Post-rut	156 to 171	15.09.2013 to 30.09.2013	Period after rutting season till end of observation period.

#### GPS data

Before release, the leading cow, the adult bull, and another adult cow were tagged with GPS-telemetry collars (GPS Plus, Vectronic Aerospace, Germany). GPS-positions of the animals were recorded individually at intervals of 60 minutes (pre-release), 30 and 20 minutes for different collars (post release) to acquire a higher resolution data set in this important phase after release. The accuracy of the GPS positions was 7.62 ± 19.44 m (average ± SD) [43]. Additionally, direct observations were made and the animals' GPS-positions were calculated trigonometrically using a handhold GPS (Garmin Etrex Venture CX), a laser distance scanner (Bushnell, Yardage Pro Sport 450), and a compass. These observations were made to check whether all non-collared animals were within the herd, to further enlarge the location data set and to observe the behavior of the animals, births, etc. All calculations were made on daily sub-samples of the data set.

We pooled all GPS-positions and direct observations when the animals did not separate from each other [40], [43], [52], [53] and regarded these data as positions of the entire herd. We controlled for separation of herd members by direct observations and by calculating the distance between the centroids of individual daily data sub-samples. If an individual was > 500 m away from the others it was defined as being apart and its positions during that period were analyzed separately.

We removed redundant data from the data set if temporal proximity between pooled GPS positions were too close (≤ 15 min). Average interval between GPS fixes was 24 ± 13.8 min (mean ± SD) with minimal/maximal intervals of 0:17 h/16:40 h. Data were subdivided into single days, counting from midnight to midnight, as European bison usually do not move during the night [39], [54]. Between ten days prior to and the first 171 days after release (2nd April to 30th September 2013) we sampled 18,471 locations (120 observations and 18,351 GPS-fixes). After removal of redundancies 12,256 data points remained.

### Daily movements and habitat utilization

We calculated the size of the areas of daily use by using a conservative measurement of 85-%-isopleths for fixed kernel (kernel h_ref_) to determine the total area and 25-%-isopleths to determine the core area of areas of daily use. Börger et al. [55] found that ten locations per month are sufficient to calculate accurate kernel home ranges, while other authors state that at least 30–50 fixes are required (simulated data) [56]. Since animals do not develop a home range over the course of a single day, we called the calculated isopleths “areas of daily use” instead of “home ranges”. Exclusive use of independent observations is not necessary when range size is estimated using kernel methods, but the whole sampling regime has to be standardized to acquire statistically reliable results. Once the sampling regime is standardized, inferences are robust to sampling variation in the number of fixes, which is especially important for ecological data [55], [56], [57].

We measured the day-to-day shift of the areas of daily use by calculating the distance between consecutive days' centroids of the daily used core areas. We measured the minimal daily walking routes of the animals by calculating a straight line between subsequent daily accumulated GPS fixes. We measured the distance between the adult bull and the herd by calculating the distance between the centroids of daily GPS fixes of the adult bull and those of the herd.

### Exploration behavior

To determine exploration rate (m/month) of the European bison, we measured the distances between the centroids of the areas of daily use of the herd and the centroid of the former enclosure. To test whether the exploration behavior was linked to specific phases (Table 2) we compared the exploration rate and the distances to areas of daily use, obtained for the periods P1-P18. We used the Friedman test [51], [58] to test for behavioral changes over time, using pairwise Wilcoxon comparison with sequential Bonferroni correction as post-hoc tests. The Friedman test is a non-parametric test for equality of medians in several repeated-measures univariate groups [51].

We calculated the 99% isopleth of the complete fixed kernel (kernel h_ref_) summer home range. To obtain an overview of the habitat used each day, we calculated in a precautionary approach the 85% isopleth for the total and the 25% isopleth for the core area to receive higher accuracy in calculating the summer home range according to [55], [59], [60], [61], [62].

### Habitat preferences

We used the location data (GPS data combined with observational data) to calculate the animals' habitat preferences within the complete summer home range. The habitat preferencee was calculated by counts of location data on a habitat type [63], [64], [65], [66], [67]. Habitat types were derived from the forestry map of the land owner. Areas not labeled with a habitat type were removed from the map. Habitat preference was tested for significant differences from a random distribution using the Chi-square test comparing numbers of locations with the area of the habitat type. We used the Jacobs' modified electivity index [68], to identify the predominately used areas. This electivity index ranges from -1 (strong avoidance) over 0 (random use) to +1 (strong preference). We calculated Bailey's confidence intervals [69] to decide whether a habitat type was significantly preferred or avoided. To test whether habitat preferences changed over time we calculated electivity indices and confidence intervals for each sub-set of location data within each phase and the overall values for the complete observation period.

### Ethics statement

The animals were housed in an 89 ha enclosure under semi-natural conditions prior to reintroduction, and were under control of the local district veterinarian. The animals were owned by the NGO “Wisent-Welt Wittgenstein e.V.”. The enclosure and project area were in private ownership of Richard, Prince of Sayn-Wittgenstein-Berleburg. No protected animals were sampled. Animal handling were conducted by the NGO “Wisent-Welt Wittgenstein”, under supervision of the district veterinarian. The permit for handling and releasing the animals was granted by a contract between the NGO “Wisent-Welt Wittgenstein e.V.”, the county government of North-Rhine Westphalia, the Bezirksregierung Arnsberg, the Landesbetrieb Wald und Holz NRW and the Wittgenstein-Berleburg'sche Rentkammer.

## Results

### Daily movements and habitat utilization

The herd significantly altered the size of the areas of daily use during the observation period (core area: χ^2^ = 58.13, df = 17, p < 0.001; total area: χ^2^ = 58.19, df = 17, p < 0.001, Fig 1C). The average core areas of daily use ranged between 10 and 26 ha. The median size of total areas covered 70 to 173 ha. Areas of daily use were largest during pre- and post-rut (Table 3). One day previous to the birth of calves on days 24 and 41 the herd increased the size of the area of daily use first and then decreased it for 10 to 20 days afterwards. The areas of daily use during the birthing phase of cows between P4 and P10 were significantly smaller than in other phases (Fig 1C, Fig 2, S1A and S1B Table). Shortly before and during the rutting season in August/September the animals increased their areas of daily use. The largest area of daily use was observed in P18 (Fig 1C, Fig 2). The size of areas of daily use in phases P12, P15 and P18 were accompanied by short excursions into new areas.

**Table 3:** Overview of measurements during the six phases. The median is shown, 1st and 3rd quartiles are given in brackets.

	Pre-release	Post-release	Birthing of cows, herd	Solitary roaming bull	Pre-rut	Rut	Post-rut
Distance to previous enclosure [m]	361 (270–376)	454 (364–1.326)	1.420 (808–2.121)	1.131 (861–1.646)	1.814 (1.054–2.293)	2.011 (1.073–2.714)	3.236 (2.921–3.708)
Areas of daily use 85% [ha]	10 (1–29)	70 (25–167)	74 (36–115)	92 (38–241)	125 (54–280)	119 (73–194)	173 (120–505)
Areas of daily use 25% [ha]	1 (0–3)	10 (2–26)	10 (5–16)	10 (6–17)	20 (8–47)	19 (11–28)	26 (15–70)
Day-to-day shift of areas used [m]	73 (55–190)	389 (18–927)	481 (269–872)	563 (231–930)	836 (531–1.314)	900 (458–1.243)	596 (298–1.273)
Distance between bull and herd [m]	4 (2–9)	108 (16–269)		2,002 (712–2.553)	168 (93–250)	119 (72–207)	90 (40–232)
Minimal daily walking routes [m]	1.487 (855–1.905)	5.072 (2.475–7.052)	3.638 (3.168–4.200)	2.514 (1.926–4.006)	5.171 (4.396–6.228)	4.349 (3.653–5.207)	4.780 (4.348–5.902)

The day-to-day shift of areas of daily use differed significantly between the 10-days-periods (χ^2^ = 61.66, df = 17, p < 0.001, S1D Table). The day-to-day shift of areas of daily use during the birthing phase (P4—P10) differed significantly from those during the pre-rut (P10—P12) and the post-rut (P17–P18, Fig 2, S1A–S1E Table). After the first exploration in P3, the day-to-day shift of areas of daily use was at median 481 m (269–872 m) during the phase of birthing (P4–P10, Fig 2, Table 3). After that phase the day-to-day shift of areas of daily use increased to 900 m (458–1,243 m) during the rut in August/September. The largest day-to-day shift of 4,609 m was recorded in P18 with highest values for the size of areas of daily use and the largest distance to the former enclosure. In general, the areas of daily use were typically not more than 1,500 m apart from that of the previous day. In general, animals showed an alternation of longer and shorter day-to-day shifts of areas of daily use every two or three days.

The length of minimal daily walking routes differed significantly between the 10-days-periods (χ^2^ = 88.13, df = 17, p < 0.001, (Fig 2, Table 3, S1E Table) and were highest during the post-release phase with a median distance of 5,072 m, and one of 5,171 m in the pre-rut phase. The longest covered route was 8,843 m on day 166. The herd's minimal daily walking distance was especially low in the first days after the two birth events with the minimum of 1,790 m occurring on day 42. The highest values of more than 11 km were shown by the herd during the first exploration phase on day 14.

The adult bull separated from the herd between day 24 and 82 in the phases P4—P10 (May—July) and was analyzed separately. He left the herd at the day of the birth of the first calf on day 24 and successively increased his distance from the herd to a median of 2,002 m (712–2,553 m, max. 4,764 m at P9, Fig 2, Table 3). The solitary adult bull's areas of daily use were only slightly larger than those of the herd (Table 3). Cows never separated from the herd. The lone roaming adult bull covered lower daily walking routes than the herd, with the exception of a few larger displacements. The minimal daily routes of the adult bull were 2,514 m in median (1,926–4,006 m). His areas of daily use were comparable in size to those of the herd with the exception of three events lasting one to three days in P7, P9 and during P10, when his areas of daily use increased up to 300% (core areas: 95 ha, 151 ha, 113 ha).

Although distances between the herd and the adult bull were large (in detail 2,211 and 2,717 m one day prior to birth), at the days of the two births the adult bull entered the herd (by reducing the distance to less than 500 m) immediately after each birth and then left the herd for a few days again. During his entire roaming phase of 58 days he reduced the distances to the herd three times for short events, and finally returned to the herd on day 82. The highest values of more than 11 km were shown by the adult bull when he searched for the herd on day 72. The herd did not show an increase in roaming activity (Fig 1C, Fig 2).

### Exploration behavior

The animals (total herd) started to explore the new habitat around day 10 (21st April) after removal of the fence on 11th April 2013. The exploration rate during the first six months was 539 m/month, calculated from average values (Table 3). The distances to the enclosure differed significantly between the 10-days-periods (χ^2^ = 101.18, df = 17, p < 0.001, Table 3, S1A–S1E Table). The first increase in the distance to the former enclosure on day 11 was 837 m, and rose further to 1,290 m on day 13 in P3 (Fig 2). A second major increase in distance to the former enclosure occurred on day 110 in P13 with an increase to 5,486 m and a third one on day 171 in P18 to 7,969 m. These maximal distances were accompanied by increases in the size of areas of daily use, increases in the day-to-day shift of used areas, and by explorations of previously unused areas (Fig 1C). The median distances between the former enclosure and the areas of daily use increased gradually and were highest with 3,236 m (2,921–3,708 m) after the rut in September 2013 (Table 3), six months after release. The animals returned to the former enclosure with the feeding site only occasionally during the six months period. All measurements mentioned above differed between the periods P1 to P18, and subsequently between the six different phases (S1A–S1E Table). Strongest differences in the rate of habitat exploration were found during pre and post release (P1—P2), at the end of the birthing phase (P8—P9) and at the end of the rut and the post-rut (P15—P18, S1A Table). The size of the overall area the herd used, the summer home range, on 30th September, 171 days after release, was 45.2 km^2^, the core area covered 1.85 km^2^ (99% and 25% kernel h_ref_, Fig 1A). We note that by the end of our observation period, the herd’s core area had grown to twice the size of the original enclosure.

### Habitat preferences

Within the complete summer home range of 45.2 km^2^, 10,468 location data were obtained (Table 4). The distribution of the animals' locations within each habitat type differed significantly from a random distribution (χ^2^ = 1,308.2, df = 10, p < 0.001). In general the animals showed preferences for spruce forest, storm damaged areas and grasslands. They avoided beech forest and other structures like settlements, functional forestry areas and pine plantations. Other forest types were frequented in relation to their size (Table 4, S2A–S2G Table).

**Table 4:** Habitat preferences in each phase and overall. Jacobs preference indices and Bailey's confidence intervals were calculated [68], [69]. Habitat types were: (+) preferred; (-) avoided; (=) used according to their size.

Habitat type	Pre-release	Post-release	Birthing / roaming bull	Pre-rut	Rut	Post-rut	Overall
Beech	**-**	**-**	**-**	**=**	**-**	**-**	**-**
Spruce	**-**	**=**	**+**	**=**	**+**	**+**	**+**
Oak	**=**	**=**	**=**	**=**	**=**	**=**	**=**
Douglas fir	**=**	**=**	**=**	**=**	**=**	**=**	**=**
Larch	**=**	**=**	**=**	**=**	**=**	**=**	**=**
Other deciduous forest	**=**	**=**	**+**	**=**	**-**	**=**	**=**
Storm damaged area	**=**	**+**	**+**	**=**	**=**	**=**	**+**
Grassland	**+**	**+**	**+**	**=**	**+**	**+**	**+**
Road	**+**	**+**	**=**	**=**	**=**	**=**	**=**
Creek	**=**	**+**	**=**	**=**	**=**	**=**	**=**
Other	**=**	**=**	**=**	**=**	**=**	**=**	**-**
Location data (n)	352	1.350	4.130	1.868	2.408	360	10.468

The distribution of the animals' locations within each habitat type was significantly different from a random distribution in all six phases (pre-release: χ^2^ = 179.69, df = 10, p < 0.001; post-release: χ^2^ = 1,237.3, df = 10, p < 0.001; birthing: χ^2^ = 474.82, df = 10, p < 0.001; pre-rut: χ^2^ = 55.052, df = 10, p < 0.001; rut: χ^2^ = 245.74, df = 10, p < 0.001; post-rut: χ^2^ = 113.05, df = 10, p < 0.001).

The animals showed clear preferences for different habitat types, except during the pre-rut phase, when no preference or avoidance could be found. These preferences, however, changed slightly during the observation period (Table 4, S2A–S2G Table). Storm damaged areas and other deciduous forest types were preferred only in spring. Spruce forests were avoided in spring, but preferred later in the year. Roads and creeks were preferred during the initial phase around release. Grasslands were continuously preferred and beech forests continuously avoided.

## Discussion

### Daily movements and habitat utilization

During the first 6 months after reintroduction of the herd, the sizes of areas of daily use found in our study were comparable to home ranges of free ranging herds in Poland [70], [71]. Home range sizes are probably most related to the availability of food [71], [72]. Monthly home range sizes in Białowieża are significantly larger during the rut than during pre-rut in mixed herds [73]. Similarly, the sizes of areas of daily use of the herd in our observation were largest before, during, and especially after the rut. During the pre- and post-rut phases the animals conducted two explorations into unknown areas. The movement of a herd, especially the start of walking, is usually determined by the leading cow. If this animal is engaged in rutting behavior it may shift its behavior to more short-term locomotion and reduced foraging [54] or resting, thereby increasing the herd's areas of daily use.

The sizes of the areas of daily use in the Rothaargebirge herd differed among phases and appeared to be strongly affected by birthing of calves. Prior to and during the birthing phase the areas of daily use of the herd were comparably small but increased just before parturition and decreased afterwards for a several days. An European bison cow may leave the herd to give birth [18], [74]. Calves remain stationary close to the place of birth for the first 1 to 20 hours of their life. Afterwards, they follow when the herd is moving [75]. In the Rothaargebirge, the first calf was born by the leading cow, which was spotted with the herd one day prior and after parturition. The second calf was born by another cow within the herd. European bison calves usually stay especially close to their mother during the first week [74], [75], [76]. Our observations suggest that the entire herd adjusted their exploration behavior to the locomotion ability of the newborn calves for about two weeks and, thus, decreased areas of daily use, the day-to-day shift and walking distances (Fig 2) [18], [74].

In the Rothaargebirge the European bison did not shift the location of daily use areas more than 1,500 m from day to day (Fig 2, Table 3). The distances fluctuated every two or three days between several hundred and more than one thousand meters. Such behavior is known for other mixed herds as well [18], [77]. Longer shifts of the areas of daily use occurred only occassionally. We could identify only four dislocations of more than 2 km, namely at the very beginning of the exploration (P3), at the beginning and end of rut (P13, P16) and at the start of the third exploration event (P18).

The animals in the Rothaargebirge roamed less than animals in other populations. The daily walking distances were, with few exceptions, more or less constant during the observation period after release. The highest values of more than 11 km were found during the first exploration phase of the herd in P3. In the calving season, the herd covered lower distances after the births, and larger distances previous to the rut. Walking distances of mixed herds are described as highly variable. They range between 2 and 17 km, changing between months and depending on phenological cycles [54], [71]. The situation of the herd in the Rothaargebirge differs from most other free roaming populations, in that no bachelor bulls or other herds were around. Nevertheless, the adult bull showed a roaming behavior that was similar to bulls of other populations. In our study the adult bull separated from the herd, immediately after the first birth for 58 days (Fig 1, Fig 2). He returned to the herd several times for only short periods during that time. After this period of absence he suddenly caught up and remained with the herd. Bulls of free ranging European bison may leave herds with the start of the calving season [71], [73], [77]. They live either alone or in bachelor groups and enter herds previous to or at the beginning of the rut in July or August [18], [73]. This behavior seems to be age dependent [18], [78]. Young European bison bulls may visit herds with estrous cows to mate even before the rutting season when older bulls are not staying with the herds [18], [79]. Adult bulls show increased roaming activity during the rut under natural population conditions and visit several herds [18], [78]. Similar behavioral patterns occur in the American bison [80], [81].

The adult bull's areas of daily use during the roaming phase were similar to those of the herd except on three occasions in P7, P9 and P10, when the adult bull contacted the herd. The lone adult bull covered lower walking distances than the herd. Similar behavior is reported from other populations: home ranges and daily searched areas of lone bulls vary between the periods of the growing seasons and from year to year, most probably related to habitat quality in terms of food [71]. The movement of the animals might be further influenced and stimulated by the movement of conspecifics, and therefore the walking routes are higher for a herd than for a lone bull.

### Exploration behavior

The reintroduced European bison continuously increased their distance from their former enclosure, by an average value of 539 m/month. The explored habitat was enlarged three times to a complete summer home range size of 45 km^2^. An animals' exploration rate is affected by several factors, including the environmental barriers, the movement pace, feeding frequency or site fidelity [70]. The main reason for exploration behavior is searching for preferred feeding habitats. Under free-ranging conditions European bison switch to patches with high herbaceous vegetation cover [71]. They leave these places after a few days, thus avoiding overgrazing of the vegetation. The use of small preferred patches in the forest, covering 100–200 ha, takes at most 7–10 days [71]. We observed a similar behavioral pattern in our European bison herd on a smaller temporal scale (Fig 2). The observed speed and pattern of exploration of the herd in the Rothaargebirge lies within the range of the exploration rate of reintroduced European bison in the Bieszczady Mountains [18], [52]. These exploration rates varied between 0.7 and 5.3 km / year [70]. The animals in our study preferred foraging sites in adjacent valleys, clear cuts and light forest with dense herbal vegetation (Fig 1B). Occasionally, the herd entered the former enclosure and the feeding site, but these events were infrequent and rare.

The exploration behavior of the Wood bison (*Bison bison athabascae*) is described as “a series of [population] increases in local areas followed by pulses of dispersal and range expansion” [82]. Our results showed that a similar behavioral pattern occurs on the level of a single herd as it explores new areas as well. In our study, the European bison explored the new habitat in three different exploration events: in the post-release, pre-rut and post-rut phase. These events were accompanied by larger areas of daily use, larger day-to-day shifts of these areas and longer walking distances. In all occasions, the animals left a previously used area and entered an area that was previously unknown to them. The distances to the former enclosure increased gradually to a maximum of almost 8 km by the third exploration event. In the first two exploratory phases (pre-rut and during rut), the animals showed higher levels of exploratory activities (daily use, day-to-day shifts, and walking distances). This difference might be a function of generally higher levels of movement associated with the rut (in terms of searching for other herds or solitary bulls) or might be due to the increased activity of the leading cow engaged in rutting behavior (see above). Within the post-rut phase the exploration activity of the herd was slightly reduced, but the distance to the former enclosure remained large. At the end of the observation period the animals showed the third and largest exploration event.

Home ranges are generally dependent on the habitat quality, group size, behavioral strategies, competitors or predators. These features are unique for every habitat and shape the species specific home range [83], [84], [85], [86], [87]. In the case of European bison in the Rothaargebirge, the complete summer home range covered 45.2 km^2^ by the end of the six months’ observation period. This home range size was comparable to other free roaming herds [11], [71], [72]. Maximum home ranges of herds in other settings cover approximately 100 km^2^ [11]. Home ranges for other herds also differed seasonally, with the largest home ranges in May and smallest in April and October [11], [71].

### Habitat preferences

We observed a shift from strong habitat preferences during the spring to a lack of habitat preference during the pre-rut. We cannot rule out whether the lack of habitat preference exhibited during the pre-rut was due to its coincidence with the second exploration event, or a feature of rutting behavior. However, the rut is a period with increased roaming activity [18], [71], [73]. Food-rich grasslands (meadows and pastures) were preferred throughout the observation period, indicating their importance as a reliable food source. The preferences for roads and creeks in the initial post-release phase might have been related to the first exploration event. When walking, animals tend to use routes easy to use like roads, frozen rivers or creeks [88], [89], [90], [91], [92].

Overall, the animals in the Rothaargebirge showed clear preferences for spruce forest, storm damaged areas and grasslands, all of which are areas with high food availability. They avoided beech forests and other areas (settlements, pine plantations, area close to settlement, maintenance and operations areas like parking spaces or storage and loading zones). The herbal biomass and available nutritional energy per hectare in the study area was unevenly distributed among the forest types. It was especially low in stands of beech and young spruce, highest values were found on storm damaged areas and grasslands [48], [71], [72], [93]. An adult European bison requires up to 19.5 kg of dry plant matter per day and feeds mostly on grasses and herbs [94], [95]. The avoidance of beech forest was congruent with findings of Kuemmerle et al. [96]. The herd also avoided pine plantations, areas close to settlements, and maintenance and operations areas.

Other studies find widely different patterns of habitat preferences for the European bison, each of which differs slightly from the patterns found in our study: European bison in Białowieża prefer deciduous forest types and avoid coniferous forests [97]. In the Carpathian mountains, they show no clear preference for any particular forest type but a strong preference for managed grasslands or select forest-dominated habitats and show preferences for mosaics of forest and grasslands [96]. In the Ukraine they prefer large, open canopied forest structures dominated by fir, spruce or mixed coniferous trees [98], [99] or prefer generally broadleaved or mixed forests [11]. Some authors suggest that the European bison is actually an open grazing specialist and, as a refugee species inhabits forests only due to a lack of naturally preferred habitats [13], [34]. In general, literature suggests that European bison shows a high variability in habitat use [13], [18], [100] and to be mostly dependent on food availability [18], [73]. Our observations appear to support this generalization. One potential implication is that European bison may be relatively flexible in their habitat use and, given sufficiently high quality forage, may be reintroduced in managed landscapes like the commercial forest in the Rothaargebirge.

In recent decades many reintroductions of European bison were conducted in Eastern Europe and Asia. In 2012, 35 populations in seven countries were introduced [11], [18], [19], but the roaming and exploration behavior after release was not recorded in as much detail as in this study. The project in the Rothaargebirge is the first one in Western Europe in a densely populated area, where the animals were released into a commercial forest with several mostly monoculture forest plantations [29]. This project is attracting considerable interest by managers of other planned reintroductions [101], [102], [103], but recently serious problems arose as the animals damaged trees of a private landlord [104], [105], [106], [107]. Our studies, however, showed the animals' ability to live in an intensively used commercial forest [45], [108]. It remains to be seen whether acceptance of the presence of the European bison will grow in this area and in Western Germany in general. It may require support on the political level to be successful.

Although we described in detail the exploration behavior of the herd after release, this study can only put a spotlight on a single herd. Therefore our findings should be interpreted cautiously, since the behavior of other populations under different conditions might differ. Nevertheless, our study gives detailed insight into the exploration behavior of this large herbivore in a human-dominated landscape back into the wild.

## Supporting Information

S1 Table Tables of Post-hoc test results. Pairwise Wilcoxon comparison with sequential Bonferroni correction. Measurements of the different 10-day periods are compared. Significant results are indicated by light gray background. Calculations are given for (a) expansion rate; (b) areas of daily use of the herd, complete area (kernel h_ref_ 85%); (c) areas of daily use of the herd, complete area (kernel h_ref_ 25%); (d) day-to-day shift of daily used areas; (e) minimal daily walking routes.
(PDF)

S2 Table Habitat preferences. For each period of higher ranking behaviour Jacobs' preference indicies for each habitat type and Bailey's confidence intervals were calculated. The area of each habitat type is given. According to the number of location counts the expected and observed location data (Expected, Observed) and the respective proportions (p(exp), p(obs)) are given. Bailey's confidence intervals (Bailey -, Bailey +) are calculated and Jacobs preference index (Jacobs). The preferences are calculated according to these borders; p(obs) lies between Bailey–and Bailey +: (=) p(exp) lies within the range of the confidence intervals, the habitat type is used according to its size. (-) p(exp) lies above the range of the confidence intervals, the habitat type is avoided. (+) p(exp) lies below the range of the confidence intervals, the habitat type is preferred.
(PDF)

S3 Table Raw location data. For each record the timestamp (UTC), Gauss-Krueger coordinates (EPSG 31467), the animal ID, the dilution and the day after release is given.
(CSV)
