# Supplementary material for: First Steps into the Wild – Exploration Behavior of European Bison after the First Reintroduction in Western Europe
Source: PLoS One. 2015 Nov 25;10(11):e0143046. doi: 10.1371/journal.pone.0143046 (PMC4659542; doi:10.1371/journal.pone.0143046)
Supplement: S1 Table — Pairwise Wilcoxon comparison with sequential Bonferroni correction. Measurements of the different 10-day periods are compared. Significant results are indicated by light gray background. Calculations are given for (a) expansion rate; (b) areas of daily use of the herd, complete area (kernel href 85%); (c) areas of daily use of the herd, complete area (kernel href 25%); (d) day-to-day shift of daily used areas; (e) minimal daily walking routes. (PDF) [file pone.0143046.s001.pdf]

Supporting information 1:

Tables of Post-hoc test results: pairwise Wilcoxon comparison with sequential Bonferroni correction. Measurements of the different 10-day periods are compared. Significant results are indicated by light gray backgroud.

Calculations are given for

a) expansion rate,
b) daily used areas of the herd, complete area (kernel h_{ref} 85%),
c) daily used areas of the herd, core area (kernel h_{ref} 25%),
d) day-to-day shift of daily used areas,
e) minimal daily walking routes.

a) expansion rate

		pre-release	post-release		birthing of cows, roaming bull						pre-rut			rut				post-rut	
		P1	P2	P3	P4	P5	P6	P7	P8	P9	P10	P11	P12	P13	P14	P15	P16	P17	P18
pre-release	P1																		
post-release	P2	0.625																	
	P3	0.003	0.003																
birthing of cows, roaming bull	P4	0.003	0.003	0.276															
	P5	0.011	0.003	0.276	0.020														
	P6	0.003	0.003	0.276	0.376	0.005													
	P7	0.011	0.003	0.922	0.376	0.276	0.233												
	P8	0.038	0.028	0.132	0.005	0.770	0.005	0.065											
	P9	0.003	0.003	0.038	0.028	0.003	0.432	0.106	0.011										
pre-rut	P10	0.003	0.003	0.323	0.323	0.085	0.922	0.011	0.005	0.865									
	P11	0.003	0.003	0.276	0.432	0.050	0.846	0.085	0.003	0.323	0.276								
	P12	0.003	0.003	0.233	0.323	0.015	0.194	0.065	0.011	1.000	0.846	0.696							
rut	P13	0.005	0.003	0.194	0.493	0.085	0.625	0.005	0.015	0.846	0.557	0.376	0.625						
	P14	0.005	0.003	0.432	0.922	0.106	0.432	0.323	0.028	0.493	0.846	1.000	0.194	0.132					
	P15	0.011	0.007	0.493	0.050	1.000	0.005	0.161	0.493	0.015	0.011	0.003	0.005	0.011	0.020				
	P16	0.003	0.003	0.020	0.028	0.003	0.085	0.020	0.003	0.922	0.696	0.050	0.770	1.000	0.132	0.003			
post-rut	P17	0.003	0.003	0.003	0.003	0.003	0.003	0.003	0.003	0.003	0.007	0.003	0.038	0.432	0.011	0.003	0.003		
	P18	0.003	0.003	0.015	0.011	0.011	0.015	0.003	0.005	0.038	0.003	0.005	0.007	0.194	0.005	0.003	0.020	0.696	

b) areas of daily use of the herd (complete area)

		P1	P2	P3	P4	P5	P6	P7	P8	P9	P10	P11	P12	P13	P14	P15	P16	P17	P18
		pre-release	post-release		birthing of cows, roaming bull						pre-rut			rut				post-rut	
pre-release	P1																		
post-release	P2	0.065																	
	P3	0.003	0.003																
birthing of cows, roaming bull	P4	0.020	0.085	0.085															
	P5	0.003	0.050	0.233	0.625														
	P6	0.015	0.028	0.050	0.696	0.432													
	P7	0.020	0.194	0.194	1.000	0.625	0.625												
	P8	0.015	0.132	0.194	0.846	0.770	0.846	1.000											
	P9	0.028	0.323	0.065	1.000	0.770	0.922	0.922	1.000										
pre-rut	P10	0.015	0.011	0.557	0.276	0.625	0.194	0.376	0.432	0.770									
	P11	0.005	0.085	0.106	1.000	0.922	0.432	0.493	1.000	0.432	0.625								
	P12	0.003	0.003	0.770	0.050	0.038	0.003	0.050	0.028	0.015	0.106	0.015							
rut	P13	0.003	0.007	1.000	0.005	0.106	0.028	0.065	0.028	0.005	0.276	0.020	0.557						
	P14	0.003	0.020	0.432	0.161	0.376	0.050	0.194	0.432	0.161	0.922	0.493	0.038	0.085					
	P15	0.003	0.020	0.233	0.625	0.922	0.233	0.557	0.557	0.696	0.625	1.000	0.038	0.065	0.557				
	P16	0.011	0.065	0.432	0.323	0.161	0.106	0.106	0.376	0.132	0.194	0.161	0.493	0.846	0.922	0.161			
post-rut	P17	0.003	0.007	0.323	0.161	0.432	0.028	0.493	0.194	0.432	0.770	0.557	0.028	0.106	0.432	0.922	0.696		
	P18	0.003	0.005	0.233	0.038	0.085	0.015	0.005	0.005	0.194	0.106	0.038	0.432	0.557	0.085	0.007	0.276	0.028	

c) areas of daily use of the herd (core area)

		P1	P2	P3	P4	P5	P6	P7	P8	P9	P10	P11	P12	P13	P14	P15	P16	P17	P18
		pre-release	post-release		birthing of cows, roaming bull						pre-rut			rut				post-rut	
pre-release	P1																		
post-release	P2	0.065																	
	P3	0.003	0.020																
birthing of cows, roaming bull	P4	0.020	0.065	0.106															
	P5	0.003	0.065	0.323	0.557														
	P6	0.015	0.065	0.132	0.493	0.625													
	P7	0.020	0.233	0.194	1.000	0.696	0.696												
	P8	0.011	0.132	0.194	0.770	0.770	1.000	0.846											
	P9	0.028	0.276	0.161	0.922	1.000	0.770	0.922	0.696										
pre-rut	P10	0.015	0.011	0.625	0.276	0.625	0.194	0.376	0.323	0.922									
	P11	0.003	0.065	0.233	0.557	1.000	0.323	0.376	0.376	0.696	0.922								
	P12	0.003	0.005	0.161	0.050	0.028	0.003	0.038	0.015	0.020	0.132	0.015							
rut	P13	0.003	0.007	0.493	0.005	0.038	0.028	0.065	0.020	0.011	0.233	0.028	0.557						
	P14	0.003	0.028	0.922	0.085	0.233	0.050	0.233	0.038	0.276	0.846	0.493	0.038	0.276					
	P15	0.003	0.020	0.432	0.493	0.922	0.233	0.323	0.323	0.770	0.625	0.696	0.020	0.065	0.557				
	P16	0.011	0.065	0.922	0.323	0.194	0.085	0.065	0.376	0.132	0.233	0.323	0.161	0.770	1.000	0.194			
post-rut	P17	0.003	0.007	0.625	0.132	0.432	0.065	0.557	0.132	0.557	1.000	0.696	0.020	0.132	0.432	0.922	0.696		
	P18	0.003	0.005	0.233	0.038	0.085	0.011	0.005	0.003	0.194	0.085	0.085	0.376	0.696	0.085	0.011	0.323	0.028	

d) day-to-day shift of daily used areas

		P1	P2	P3	P4	P5	P6	P7	P8	P9	P10	P11	P12	P13	P14	P15	P16	P17	P18
		pre-release	post-release		birthing of cows, roaming bull						pre-rut			rut				post-rut	
pre-release	P1																		
post-release	P2	0.085																	
	P3	0.011	0.038																
	P4	0.015	0.065	0.432															
birthing of cows, roaming bull	P5	0.005	0.028	0.233	0.846														
	P6	0.003	0.038	0.493	0.770	0.557													
	P7	0.005	0.015	0.276	0.922	0.493	0.922												
	P8	0.015	0.050	0.276	0.696	0.557	1.000	0.493											
	P9	0.015	0.028	0.696	0.557	0.233	0.557	0.557	0.696										
pre-rut	P10	0.005	0.003	1.000	0.276	0.020	0.007	0.085	0.161	0.276									
	P11	0.003	0.005	0.770	0.625	0.038	0.161	0.276	0.625	1.000	0.493								
	P12	0.003	0.005	0.625	0.050	0.003	0.065	0.065	0.020	0.106	0.132	0.050							
rut	P13	0.003	0.003	0.493	0.020	0.011	0.003	0.015	0.050	0.020	0.233	0.106	1.000						
	P14	0.003	0.007	0.922	0.065	0.020	0.132	0.085	0.020	0.050	0.557	0.194	0.696	0.557					
	P15	0.003	0.007	0.846	0.376	0.065	0.065	0.132	0.323	0.922	0.323	0.376	0.276	0.161	0.233				
	P16	0.003	0.011	0.770	0.323	0.028	0.085	0.106	0.376	0.323	0.922	0.276	0.846	0.625	0.696	0.376			
post-rut	P17	0.038	0.194	0.038	0.233	0.432	0.028	0.194	0.194	0.085	0.003	0.050	0.007	0.003	0.015	0.015	0.020		
	P18	0.005	0.007	0.696	0.132	0.028	0.106	0.085	0.161	0.557	0.376	0.493	0.770	0.846	1.000	0.625	0.846	0.028	

e) daily minimum walking routes

		P1	P2	P3	P4	P5	P6	P7	P8	P9	P10	P11	P12	P13	P14	P15	P16	P17	P18
		pre-release	post-release		birthing of cows, roaming bull						pre-rut			rut				post-rut	
pre-release	P1																		
post-release	P2	0.015																	
	P3	0.003	0.003																
birthing of cows, roaming bull	P4	0.003	0.038	0.011															
	P5	0.003	0.003	0.011	0.770														
	P6	0.003	0.028	0.005	0.376	0.065													
	P7	0.003	0.050	0.003	0.161	0.015	1.000												
	P8	0.003	0.028	0.005	0.846	0.233	0.557	0.323											
	P9	0.003	0.085	0.005	0.233	0.132	0.922	0.770	0.625										
pre-rut	P10	0.003	0.003	0.015	0.432	0.194	0.015	0.003	0.005	0.011									
	P11	0.003	0.007	0.011	0.770	1.000	0.065	0.085	0.194	0.020	0.233								
	P12	0.003	0.003	0.085	0.161	0.161	0.003	0.003	0.015	0.005	0.696	0.028							
rut	P13	0.003	0.007	0.011	0.161	0.493	0.020	0.011	0.028	0.011	0.846	0.050	0.770						
	P14	0.003	0.028	0.007	0.557	0.557	0.085	0.028	0.493	0.028	0.132	0.493	0.028	0.038					
	P15	0.003	0.007	0.011	0.625	0.132	0.770	0.922	0.922	1.000	0.050	0.323	0.015	0.065	0.323				
	P16	0.003	0.007	0.011	0.770	0.922	0.050	0.005	0.161	0.015	0.085	0.557	0.065	0.194	0.376	0.194			
post-rut	P17	0.003	0.005	0.005	1.000	0.376	0.106	0.020	0.106	0.132	0.015	0.432	0.007	0.085	0.846	0.432	0.376		
	P18	0.003	0.003	0.323	0.376	0.132	0.011	0.005	0.028	0.011	0.696	0.132	0.846	0.770	0.106	0.003	0.432	0.028	
