# Supplementary material for: First Steps into the Wild – Exploration Behavior of European Bison after the First Reintroduction in Western Europe
Source: PLoS One. 2015 Nov 25;10(11):e0143046. doi: 10.1371/journal.pone.0143046 (PMC4659542; doi:10.1371/journal.pone.0143046)
Supplement: S2 Table — For each period of higher ranking behaviour Jacobs' preference indicies for each habitat type and Bailey's confidence intervals were calculated. The area of each habitat type is given. According to the number of location counts the expected and observed location data (Expected, Observed) and the respective proportions (p(exp), p(obs)) are given. Bailey's confidence intervals (Bailey -, Bailey +) are calculated and Jacobs preference index (Jacobs). The preferences are calculated according to these borders; p(obs) lies between Bailey–and Bailey +: (=) p(exp) lies within the range of the confidence intervals, the habitat type is used according to its size. (-) p(exp) lies above the range of the confidence intervals, the habitat type is avoided. (+) p(exp) lies below the range of the confidence intervals, the habitat type is preferred. (PDF) [file pone.0143046.s002.pdf]

Supporting information 2:

For each period of higher ranking behaviour Jacobs' preference indicies for each habitat type and Bailey's confidence intervals were calculated.
The area of each habitat type is given.
According to the number of location counts the expected and observed location data (Expected, Observed)
and the respective proportions (p(exp), p(obs)) are given.
Bailey's confidence intervals (Bailey -, Bailey +) are calculated and Jacobs preference index (Jacobs).

The preferences are calculated according to these borders; p(obs) lies between Bailey – and Bailey +:

(=) p(exp) lies within the range of the confidence intervals, the habitat type is used according to its size.
(-) p(exp) lies above the range of the confidence intervals, the habitat type is avoided.
(+) p(exp) lies below the range of the confidence intervals, the habitat type is preferred.

a) pre-release

Habitat type	Area m²	Expected	Observed	p(exp)	p(obs)	Bailey -	Bailey +	Jacobs	Preference
Beech	16805779	147	72	0.4164	0.2045	0.1459	0.2699	-0.4702	-
Spruce	16577187	145	110	0.4108	0.3125	0.2428	0.3849	-0.2106	-
Oak	8118	0	0	0.0002	0.0000	0.0000	0.0156	-1.0000	=
Douglas fir	95643	1	0	0.0024	0.0000	0.0000	0.0156	-1.0000	=
Larch	44433	0	0	0.0011	0.0000	0.0000	0.0156	-1.0000	=
Other deciduous forest	220585	2	0	0.0055	0.0000	0.0000	0.0156	-1.0000	=
Wind thrown areas	1097805	10	5	0.0272	0.0142	0.0018	0.0413	-0.3199	=
Greenland	2659188	23	103	0.0659	0.2926	0.2246	0.3641	0.7086	+
Road	2696086	24	62	0.0668	0.1761	0.1213	0.2387	0.4983	+
Creek	34391	0	0	0.0009	0.0000	0.0000	0.0156	-1.0000	=
Other	118950	1	0	0.0029	0.0000	0.0000	0.0156	-1.0000	=

**b) post-release**									
Habitat type	Area m²	Expected	Observed	p(exp)	p(obs)	Bailey -	Bailey +	Jacobs	Preference
Beech	16805779	562	170	0.4164	0.1259	0.1012	0.1531	-0.6640	-
Spruce	16577187	555	504	0.4108	0.3733	0.3357	0.4111	-0.0784	=
Oak	8118	0	0	0.0002	0.0000	0.0000	0.0041	-1.0000	=
Douglas fir	95643	3	7	0.0024	0.0052	0.0011	0.0132	0.3739	=
Larch	44433	1	0	0.0011	0.0000	0.0000	0.0041	-1.0000	=
Other deciduous forest	220585	7	16	0.0055	0.0119	0.0049	0.0225	0.3715	=
Wind thrown areas	1097805	37	76	0.0272	0.0563	0.0397	0.0760	0.3617	+
Greenland	2659188	89	424	0.0659	0.3141	0.2783	0.3506	0.7330	+
Road	2696086	90	142	0.0668	0.1052	0.0825	0.1305	0.2430	+
Creek	34391	1	10	0.0009	0.0074	0.0022	0.0164	0.7949	+
Other	118950	4	1	0.0029	0.0007	0.0000	0.0057	-0.5990	=

**c) birth giving**									
Habitat type	Area m²	Expected	Observed	p(exp)	p(obs)	Bailey -	Bailey +	Jacobs	Preference
Beech	16805779	1720	1205	0.4164	0.2918	0.2717	0.3121	-0.2680	-
Spruce	16577187	1696	1803	0.4108	0.4366	0.4145	0.4585	0.0528	+
Oak	8118	1	0	0.0002	0.0000	0.0000	0.0013	-1.0000	=
Douglas fir	95643	10	5	0.0024	0.0012	0.0002	0.0036	-0.3243	=
Larch	44433	5	3	0.0011	0.0007	0.0000	0.0028	-0.2052	=
Other deciduous forest	220585	23	40	0.0055	0.0097	0.0058	0.0147	0.2805	+
Wind thrown areas	1097805	112	205	0.0272	0.0496	0.0404	0.0599	0.3026	+
Greenland	2659188	272	612	0.0659	0.1482	0.1327	0.1643	0.4230	+
Road	2696086	276	255	0.0668	0.0617	0.0515	0.0730	-0.0421	=
Creek	34391	4	2	0.0009	0.0005	0.0000	0.0023	-0.2755	=
Other	118950	12	0	0.0029	0.0000	0.0000	0.0013	-1.0000	-

d) pre-rut									
Habitat type	Area m²	Expected	Observed	p(exp)	p(obs)	Bailey -	Bailey +	Jacobs	Preference
Beech	16805779	778	761	0.4164	0.4074	0.3748	0.4398	-0.0186	=
Spruce	16577187	767	800	0.4108	0.4283	0.3954	0.4609	0.0359	=
Oak	8118	0	0	0.0002	0.0000	0.0000	0.0030	-1.0000	=
Douglas fir	95643	4	12	0.0024	0.0064	0.0022	0.0134	0.4626	=
Larch	44433	2	2	0.0011	0.0011	0.0000	0.0052	-0.0140	=
Other deciduous forest	220585	10	4	0.0055	0.0021	0.0002	0.0070	-0.4384	=
Wind thrown areas	1097805	51	36	0.0272	0.0193	0.0112	0.0299	-0.1746	=
Greenland	2659188	123	147	0.0659	0.0787	0.0618	0.0977	0.0954	=
Road	2696086	125	105	0.0668	0.0562	0.0419	0.0727	-0.0917	=
Creek	34391	2	0	0.0009	0.0000	0.0000	0.0030	-1.0000	=
Other	118950	6	1	0.0029	0.0005	0.0000	0.0042	-0.6932	=

e) rut									
Habitat type	Area m²	Expected	Observed	p(exp)	p(obs)	Bailey -	Bailey +	Jacobs	Preference
Beech	16805779	1003	709	0.4164	0.2944	0.2681	0.3212	-0.2620	-
Spruce	16577187	989	1171	0.4108	0.4863	0.4570	0.5151	0.1518	+
Oak	8118	0	4	0.0002	0.0017	0.0001	0.0055	0.7843	=
Douglas fir	95643	6	9	0.0024	0.0037	0.0010	0.0086	0.2246	=
Larch	44433	3	0	0.0011	0.0000	0.0000	0.0023	-1.0000	=
Other deciduous forest	220585	13	3	0.0055	0.0012	0.0000	0.0048	-0.6300	-
Wind thrown areas	1097805	66	56	0.0272	0.0233	0.0153	0.0332	-0.0802	=
Greenland	2659188	159	283	0.0659	0.1175	0.0994	0.1370	0.3075	+
Road	2696086	161	168	0.0668	0.0698	0.0557	0.0855	0.0233	=
Creek	34391	2	0	0.0009	0.0000	0.0000	0.0023	-1.0000	=
Other	118950	7	5	0.0029	0.0021	0.0003	0.0061	-0.1738	=

f) post-rut

Habitat type	Area m²	Expected	Observed	p(exp)	p(obs)	Bailey -	Bailey +	Jacobs	Preference
Beech	16805779	150	72	0.4164	0.2000	0.1425	0.2642	-0.4811	-
Spruce	16577187	148	196	0.4108	0.5444	0.4663	0.6172	0.2632	+
Oak	8118	0	0	0.0002	0.0000	0.0000	0.0153	-1.0000	=
Douglas fir	95643	1	1	0.0024	0.0028	0.0000	0.0214	0.0794	=
Larch	44433	0	0	0.0011	0.0000	0.0000	0.0153	-1.0000	=
Other deciduous forest	220585	2	0	0.0055	0.0000	0.0000	0.0153	-1.0000	=
Wind thrown areas	1097805	10	8	0.0272	0.0222	0.0054	0.0528	-0.1033	=
Greenland	2659188	24	51	0.0659	0.1417	0.0928	0.1993	0.4012	+
Road	2696086	24	32	0.0668	0.0889	0.0505	0.1381	0.1536	=
Creek	34391	0	0	0.0009	0.0000	0.0000	0.0153	-1.0000	=
Other	118950	1	0	0.0029	0.0000	0.0000	0.0153	-1.0000	=

g) Sum

Habitat type	Area m²	Expected	Observed	p(exp)	p(obs)	Bailey -	Bailey +	Jacobs	Preference
Beech	16805779	4359	2989	0.4164	0.2855	0.2730	0.2982	-0.2820	-
Spruce	16577187	4300	4584	0.4108	0.4379	0.4241	0.4517	0.0555	+
Oak	8118	2	4	0.0002	0.0004	0.0000	0.0013	0.3103	=
Douglas fir	95643	25	34	0.0024	0.0032	0.0019	0.0051	0.1567	=
Larch	44433	12	5	0.0011	0.0005	0.0001	0.0014	-0.3951	=
Other deciduous forest	220585	57	63	0.0055	0.0060	0.0041	0.0084	0.0484	=
Wind thrown areas	1097805	285	386	0.0272	0.0369	0.0318	0.0424	0.1558	+
Greenland	2659188	690	1620	0.0659	0.1548	0.1448	0.1650	0.4438	+
Road	2696086	699	764	0.0668	0.0730	0.0659	0.0804	0.0475	=
Creek	34391	9	12	0.0009	0.0011	0.0004	0.0024	0.1474	=
Other	118950	31	7	0.0029	0.0007	0.0001	0.0017	-0.6308	-
